# Illumina Synthetic Long Read Sequencing Allows Recovery of Missing Sequences even in the “Finished” *C. elegans* Genome

**DOI:** 10.1038/srep10814

**Published:** 2015-06-03

**Authors:** Runsheng Li, Chia-Ling Hsieh, Amanda Young, Zhihong Zhang, Xiaoliang Ren, Zhongying Zhao

**Affiliations:** 1 Department of Biology, Hong Kong Baptist University, 224 Waterloo Road, Kowloon Tong, Hong Kong, China; 2 Illumina Inc., 5200 Illumina Way, San Diego, 92122, USA; 3 State Key Laboratory of Environmental and Biological Analysis, Hong Kong Baptist University, Hong Kong, China

## Abstract

Most next-generation sequencing platforms permit acquisition of high-throughput DNA sequences, but the relatively short read length limits their use in genome assembly or finishing. Illumina has recently released a technology called Synthetic Long-Read Sequencing that can produce reads of unusual length, i.e., predominately around 10 Kb. However, a systematic assessment of their use in genome finishing and assembly is still lacking. We evaluate the promise and deficiency of the long reads in these aspects using isogenic *C. elegans* genome with no gap. First, the reads are highly accurate and capable of recovering most types of repetitive sequences. However, the presence of tandem repetitive sequences prevents pre-assembly of long reads in the relevant genomic region. Second, the reads are able to reliably detect missing but not extra sequences in the *C. elegans* genome. Third, the reads of smaller size are more capable of recovering repetitive sequences than those of bigger size. Fourth, at least 40 Kbp missing genomic sequences are recovered in the *C. elegans* genome using the long reads. Finally, an N50 contig size of at least 86 Kbp can be achieved with 24×reads but with substantial mis-assembly errors, highlighting a need for novel assembly algorithm for the long reads.

The high-throughput sequencing technology, also referred to as Next Generation Sequencing (NGS), has transformed biomedical research from genetics to developmental biology. Capacity of generating large volume of sequencing reads in a short period of time enables genome assembly, genotyping, expression profiling and systematic identification of DNA binding sites in a way that is difficult or impractical otherwise. One critical issue associated with all NGS platforms is the read length on top of the read throughput and accuracy. The relatively short length of sequencing reads produced by most NGS platforms^1^ limits their use particularly in genome assembly and finishing. For example, ambiguity often remains when the short reads are mapped against a reference genome or among one another, which is further complicated by the accuracy of read sequence. The short length also makes it problematic in variation call and *de novo* genome assembly. Despite the substantial efforts that have been made in the past decade to increase the read length, for example, from 22 bp to up to 300 bp by Illumina platform^2^, these lengths are still unsatisfactory for many applications, including *de novo* genome assembly, genome gap finishing and identification of complex structural variations in a draft genome. Therefore, a tradeoff has to be made across different NGS platforms to balance the read length and yield. For example, the 454 platform can produce reads of length up to 1 Kbp, which is useful in resolving genomic gaps^3^. Such tradeoff has also catalyzed the third generation sequencing, a term coined for a sequencing method capable of producing reads of unusual length. One example is the single molecule real time sequencing (SMRT) from PacBio, which is able to generate sequencing reads up to 30 Kbp and has been demonstrated to be useful in resolving the complex genomic regions^4^. However, most of the single-pass reads suffer from a high error rate up to 15-18%^5^, thus need to be corrected before being used for genome assembly and other applications. The high false positive rate of indels (insertion and deletion) also hinders the use of the PacBio reads in variation calling^5^.

Illumina has recently released Synthetic Long-Read technology (http://www.illumina.com/products/truseq-synthetic-long-read-kit.ilmn), which allows construction of synthetic long reads from the short sequencing reads generated with its existing HiSeq platform. A surprising long read length plus its high accuracy is posed to affect *de novo* genome assembly or gap finishing in a draft genome. This technology, also known as Moleculo, has been demonstrated its use by performing *de novo* genome assembly of the *Botryllus schlosseri*, a star ascidian animal^6^ and whole-genome haplotyping of humans^7^. The long reads have also been used to resolve a subset of highly-repetitive transposons in the genome of *Drosophila melanogaster*^8^. However, a systematic evaluation of the reads on their ability in resolving repetitive sequences and identification of complex genomic variations remains to be seen. In particular, the deficiency of the reads in genome finishing and *de novo* genome assembly has not been characterized. To this end, a high quality of “finished” genome that contains all resolved repetitive sequences will be needed. *C. elegans* genome is the choice for this task due to its following characteristics. First, its genome is a “finished” one with no gap^9^, providing an opportunity for unbiased evaluation of the read accuracy and genomic coverage. Second, all types of repetitive sequences have been unambiguously resolved, allowing systematic assessment of the long reads in recovering various repeats. Third, a well annotated *C. elegans* genome alone with enormous amount of NGS sequencing data permits validation of the potential errors either in the current genome assembly or in the long reads. Finally, the isogenic *C. elegans* genome suffers little heterozygosity, which is often the issue associated with *de novo* assembly and variation calling in the genomes of outcrossing organisms. By mapping the long reads and its assembled contigs back to the *C. elegans* genome, we systemically characterized the promise and deficiency of the reads in genome improvement and *de novo* genome assembly.

## Results

### Read accuracy across its length

Given that the synthetic long reads (hereafter referred to as long reads) were assembled from the short reads generated with Illumina HiSeq, only the reads longer than 1500 bp in sizes were retained for all the analysis. A total of 373,121 long reads were generated with two libraries, which covered approximately 24×of *C. elegans* genome. To filter out the possible contaminations of sequence, all reads were aligned against the NCBI non-redundant genome database by BLASTN. 195 reads were found to be contaminated ones (See Methods) and excluded from further analysis (Supplementary Table S1; Supplementary Figure S1).

**Figure 1:**
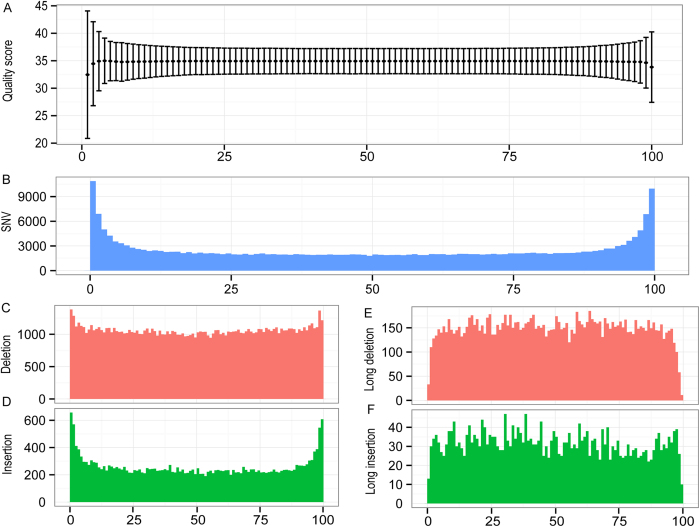
Read quality and mappability across its lengths. (**A**) Read quality score (mean ± SD). (**B**) SNV count. (**C** and **D**) Count of deletion (red) or insertion (green) respectively. (**E** and **F**) Count of long deletion (red) or insertion (> = 9 bp) (green) respectively (see Methods). Scale in horizontal axis was normalized to a 100% of the read length. The window size of the plot is 1% of the read length.

To evaluate the sequence accuracy of the long reads, we mapped all the decontaminated reads against the reference genome and calculated the ratio of sequence variations. We defined the variation as a single nucleotide variation (SNV), an insertion or deletion (indel) in the long reads relative to the reference genome. The overall ratios of SNV, insertion and deletion out of the total sequences concerned are 0.011%, 0.019% and 0.101%, respectively, indicating a high degree of sequence accuracy in the long reads. The ratios may be overestimated if they all are treated as read errors because the ratios represent the combined error rate from both reads and the reference genome. For example, a newly acquired variation through spontaneous mutation in the sequenced strain will contribute to the reference error. Notably, the ratio of SNV is much lower than the error rate of Illumina raw reads (~0.6%)^10^,^11^, which is likely due to the fact that the long-reads were built from the consensus sequence of the underlying Illumina HiSeq reads that were subjected to self-correction during pre-assembly of the long reads. SNVs and indels that are small in sizes (<9 bps, a default cutoff set by CLC Genomic Workbench) were enriched in both ends of the reads, consistent with a lower quality score in the sequences of these regions (Fig. 1).This could be due to adapter trimming or a fewer number of short reads used to build the consensus sequence at the end of long reads than that used for the central region. Most of the identified indels are relatively short in size (<9 bp) (Fig. 1C,D). However, the density of long indels (> = 9 bp) appears to be constant across read length (Fig. 1E,F). Surprisingly, the deletion rate was observed at a much higher frequency than that for SNV and insertion. All kinds of variation are relatively enriched in the repetitive regions, but depleted in the conserved regions (data not shown).

### Characteristics of read length and its impact on read mappability

**Figure 2:**
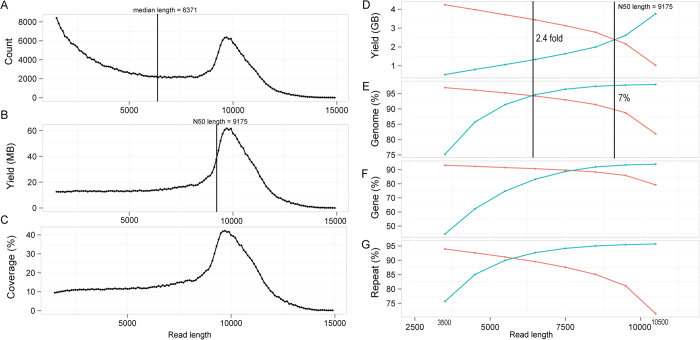
Read count and mappability across its lengths. (**A**, **B** and **C**) Count, yield and genomic coverage of long read across its lengths respectively with a window size of 100 bp. (**D**, **E**, **F** and **G**) Accumulative read yield and its coverage of genome, protein-coding genes and repetitive sequences across read lengths respectively. Blue and red lines represent accumulative yield or percentage respectively using the reads shorter or longer than a given read length.

To examine whether the reads of different sizes show differential coverage of the *C. elegans* genome, all the decontaminated long reads were split into 139 subsets with an increment of 100 bp except for those longer than 15.4 Kbp that were merged into the last subset. Reads in all the subsets were subsequently mapped against the *C. elegans* genome. Median and N50 length of the reads are 6371 and 9175 bp respectively (Fig. 2A,B) with the maximum length of 23 Kbp (data not shown). Plotting of the count versus the size of the long reads demonstrated two peaks with one centering around 1.5 Kbp and the other around 10 Kbp (Fig. 2A). Reads of around 10 Kbp suggest a full reconstruction of the DNA insert inside the library (note the insert size for the long read library is approximately 10 Kbp, see Methods). Though most reads were pre-assembled with a remarkable length of around 10 Kbp, a considerable number of shorter reads existed for which the yields and genomic coverage showed uniform distribution from 1.5 ~ 8 Kbp (Fig. 2B,C).

To evaluate the accumulative genomic coverage by the reads of a given length, reads shorter or longer than this length was pooled and mapped back to the reference genome (Figs. 2D to 2G). The accumulative reads shorter than 10.5 Kbp cover most of the reference genome, indicating that the sequences of reads longer than 10.5 Kbp are mainly redundant with those of the shorter reads. The N50 length is the demarcation that the summed yields of all reads longer and shorter than this length are equal to each other. Intriguingly, the reads shorter than the N50 cover about 7% more genome than those longer than the N50 (Fig. 2D, E). The reads shorter or longer than 6.5 Kbp produced the equal mapping coverage (~94%) of the genome (Fig. 2E), whereas the sequence yield of the reads longer than 6.5 Kbp was 2.4 times of that for the reads shorter than this size, indicating that shorter reads can recover various genomic parts with a higher efficiency. To examine how the short reads can outperform the long ones in recovering genome sequences, we investigated the capacity of the two types of reads in recovering sequences of protein-coding genes and non-coding repetitive regions respectively by their accumulative reads. The results show that accumulative reads shorter or longer than 7.5 Kbp can recover the same portion of (~90%) genes (Fig. 2F) while the intersection point for recovering the same portion of repetitive sequences is around 5.5 Kbp (Fig. 2G), indicating that the reads of smaller sizes are more capable of recovering repetitive sequences than those of bigger sizes.

To further examine the impact of read length on its genomic coverage across chromosome, we plotted the coverage of reads with different lengths across chromosomes. Interestingly, the reads with relatively short length tend to concentrate more on the two arms than the mid-part of the chromosome (Supplementary Figure S2A). Consistent with this, the arm regions of *C. elegans* autosomes is known to contain a higher density of SNPs and repetitive sequences^9^, indicating that the shorter reads are more capable of recovering repetitive sequences than the longer reads (Supplementary Figure S2A). Taken together, our data demonstrated that the long reads were able to recover most sequences of the *C. elegans* genome, and those with relatively small sizes were more efficient in recovering repetitive sequences than those with relatively big sizes.

### Detailed characterization of the long reads’ capability in recovering various repetitive sequences

One of the major challenges in genome assembly is the presence of many types of repetitive sequences that prevent assembly of a complete genome. To evaluate the capacity of the long reads in recovering the resolved repetitive sequences in the *C. elegans* genome, the long reads were mapped against multiple types of repetitive sequences as defined by Repeatmasker (http://www.repeatmasker.org) (see Methods).

**Figure 3:**
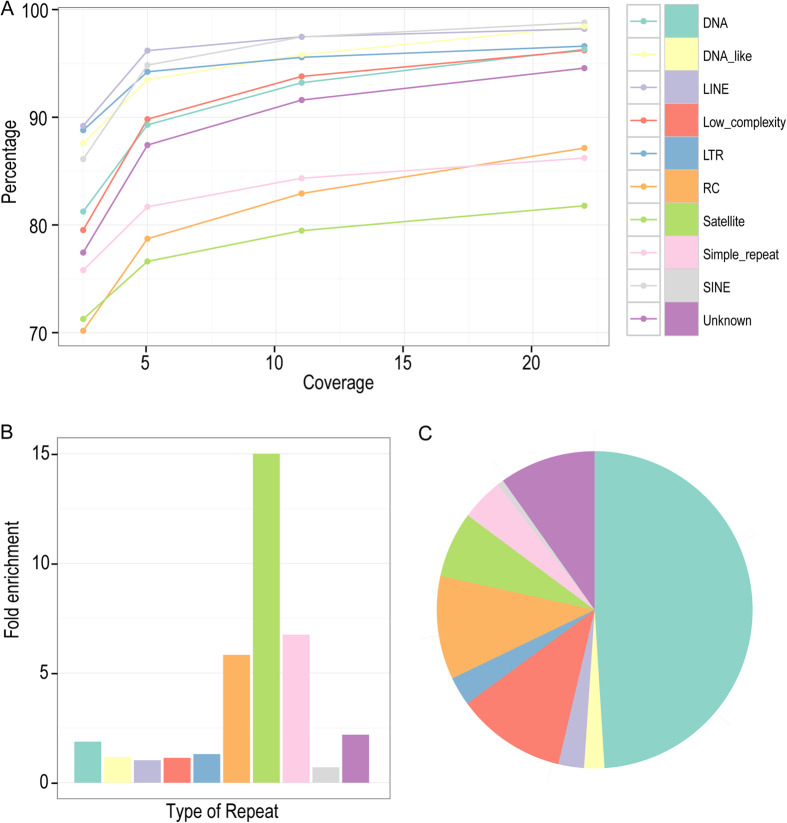
Capacity of long reads in recovering various repetitive sequences. (**A**) Effect of read coverage on the recovery rate of various types of repetitive sequence as color coded. (**B** and **C**) Relative fold enrichment and composition of various repetitive sequences within the gap regions (the genomic region not covered by any read) respectively. Same color coding scheme is used in (**A**, **B** and **C**).

Surprisingly, over 95% of various repetitive sequences can be recovered by a 24 × coverage of long reads except the satellite, RC (Helitron) and simple repeats, which can still be recovered at a ratio of approximately 87%, 86% and 82% respectively (Fig. 3A). To investigate what types of sequences are responsible for the remaining gaps that bear no coverage by any reads, we examined the sequence content within various gaps especially those that are relatively large in size. Scanning the sequences within the gaps against different type of repetitive sequences revealed that three types of repetitive sequences, satellite, RC, and simple repeat, were the most enriched repetitive sequences (Fig. 3B; Supplementary Table S5) within the gaps with the DNA repeat contributing most to the yield (Fig. 3C).

Consistent with tis, the three types of the most enriched repetitive sequences also showed the lowest recovery rate by the long reads (Fig. 3A). To further understand the likely cause underlying the observed gap region, we first examined the longest gap (~47 Kbp) left by the long reads that is located in the middle of chromosome III (Supplementary Figure S2B, S2C; Supplementary Table S5). A close examination of the sequence content within the gap revealed that the region was mainly comprised of tandem repeat units called satellite repeats “CeRep59”. The highly tandem arrangement of the repetitive sequences possibly prevents the short reads from being placed in this region as described below (Supplementary Figure S2C).

### Detailed characterization of the sequence contents within genomic gaps left by the long reads

**Figure 4:**
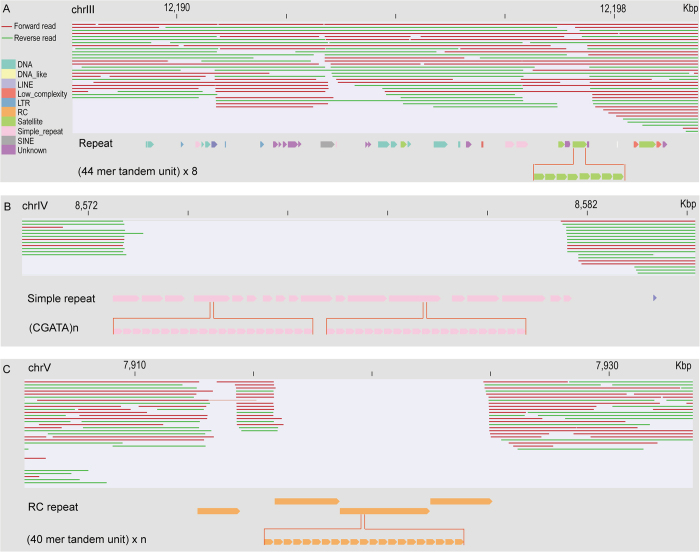
Impact of the arrangement of repetitive sequences in the reference genome on its coverage by the long reads. (**A**) An example showing the full recovery of a 10 Kbp genomic region containing various types of repetitive sequences indicated by arrows and differentially color coded by long reads. One satellite repeat within the region is highlighted for its tandem-arranged unit at the bottom. Note, certain reads cannot cover the repetitive region while the others can. (**B** and **C**) Examples of the uncovered region (gap) containing simple and RC type of repeat respectively. Note that both repetitive sequences consist of clusters formed by identical tandem units as indicated and the clusters are longer than 500 bp in size while all the repeat clusters in panel (**A**) are much smaller than this size. Tandem units are highlighted in the bottom. Same color coding scheme is used in (**A**, **B** and **C**). Chromosome coordinates are indicated on the top.

To examine why the long reads are capable of recovering genomic regions containing certain types repetitive sequences but not those carrying other types of repetitive sequences, we chose to focus on two types of genomic regions both rich in repetitive sequences, one of which was successfully recovered by the long reads (Fig. 4A) while the other failed to be recovered by any reads (Fig. 4B,C). Characterization of sequence contents within these regions will also provide insight into the issues associated with pre-assembly of the long reads to sufficient length. To this end, we first focused on a genomic region of approximately 10 Kbp in size that contains numerous types of annotated repeats, but are fully recovered by the long reads (Fig. 4A). Notably, various types of repeats are present within the region, but they show mixed distribution against one another. In addition, all of the sequence clusters formed by any single repeat-unit are shorter than 500 bp, a size with relatively low copy number that is much smaller than that of the long reads. We next concentrated on another two genomic regions around 10 and 20 Kbp in size respectively because both regions contain a 9-Kbp gap, which bears no coverage by any long read. Examination of their sequence content revealed that the former was almost entirely comprised of a single type of simple repeat (with a repeat unit of “TGATA”) (Fig. 4B) while the latter was composed of nearly all RC repeats, both of which are arranged in tandem (Fig. 4C). It is worth noting that the sizes for many clusters formed by the two types of tandem repeats are bigger than 500 bp, suggesting that it is the tandem arrangement of a single type of repeat sequence forming a cluster that is longer than around 500 bp in size that prevents pre-assembly of a long read with sufficient length (>1.5 Kbp). Therefore, a genomic region rich in these types of repetitive sequences that are arranged in a long tandem stretch (>500 bp) will not be covered by the long reads.

Notably, a single genomic region of roughly 7.2 Kb in size was recovered with unusual depth up to 594×. It is located at the end of chromosome I, within which a single copy of the 18S/5.8S/28S rDNA unit is placed in the *C. elegans* reference genome (Supplementary Figure S3A). The coverage of synthetic long read is almost uniform with respect to GC-content across the whole genome. Because the raw short reads used to generate the long reads have been normalized before pre-assembly into long read. Only the region with extreme GC content bears lower coverage than average, especially when it contains some tandem repeats. As for the genomic region of 18S/5.8S/28S rDNA unit, with about 47% GC content, the copy number can be estimated roughly by simply dividing the read coverage in this region by that of the genome average. We estimate there are approximate 27 copies of the rDNA gene within the *C. elegans* genome, which is lower than the previous estimation, i.e., ~55 copies^12^. Contrary to the rDNA cluster on the chromosome I, the 5S rDNA genic region currently placed on the chromosome V was barely covered by any reads (Supplementary Figure S3B) though about 110 copies of the gene were estimated to be present in the *C. elegans* genome^13^. The rDNA genes are expected to be arranged in tandem in the *C. elegans* genome^12^,^14^. It remains possible that the 5S rDNA gene forms extremely tandem repeats that prevents the pre-assembly of the long reads in the relevant genomic regions. Given the capability of the long read to recover the tandem repeats formed by the 18S/5.8S/28S rDNA genes with a repetitive unit of around 7.2 Kbp in size but not those formed by a small-sized repetitive unit, for example satellite, RC or simple repeat and 5S rDNA, the results indicate that the sequence complexity plays a key role in genome recovery rate by the long reads. Sequences with low complexity, for example those composed of short repeats arranged in tandem, will prevent pre-assembly of a long read with the HiSeq raw reads located within the region, leading to a gap left by the long reads. Therefore the genomic regions consisting of tandem repeats with a small-sized repetitive unit are beyond the reach of the long reads. In addition, the coverage of mitochondrial genome by the long reads was relatively low (with only 21 reads mapped) in contrast to the high mitochondrial DNA copy number^15^, which could be due to the DNA shearing and size selection in long-read library preparation, which demands 10 Kbp in size.

### Identification of the relatively long indels within reads

It is surprising that only 710 out of the 11672 SNVs identified with the long reads (Supplementary Table S3) are overlapping with those listed in the SNP database (Wormbase WS242)^16^, 681 of which are identical between each other. Consistent with this, 140 out of the 681 SNVs are overlapping with those identified using LSJ1 strain, a sister strain of N2^17^, suggesting that these SNVs are possible errors in the reference genome. There are 955 single nucleotide substitution in the *C. elegans* reference genome, which was annotated as possible reference errors using the existing RNA-seq data derived from mRNA^16^. 200 out of 955 substitutions are also observed with our SNV data. It has yet to be determined whether the remaining SNVs identified with the long reads represent the errors in reference genome, the read errors or the spontaneous mutations accumulated in our lab strain since its separation with the N2 that was used to produce *C. elegans* reference genome.

**Figure 5:**
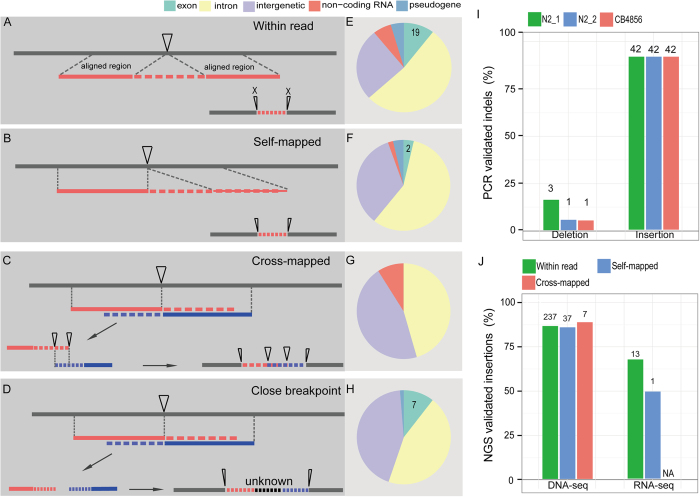
Identification and validation of the long indels (>=9 bp) by PCR and/or NGS data. (**A**-**D**) Shown are the definitions of four possible ways of detecting insertion (insertion position indicated with an inverse triangle). (**A**) “Within read” type of insertion is defined as an unalignable region within a read against N2 genome while both arms of the read can be successfully aligned with the same reference genome. (**B**) “Self-mapped” type of insertion is similarly defined as that in (**A**) except that a second arm can only be aligned with the N2 genome using more permissive alignment parameters (see Methods). (**C**) “Cross-mapped” type of insertion is defined as an insertion recovered by the overlapping unalignable ends from two independent reads located adjacently in the reference genome. Both unalignable ends that can be partially aligned against N2 genome that are adjacent to each other. (**D**) “Close breakpoint” type of insertion is similarly defined as that in (**C**) except that the recovered insertion contains a gap with unknown length due to non-overlapping part between the two unalignable ends. (**E**-**H**) Genomic distribution of each type of insertion identified in (**A**-**D**). Genomic regions are classified based on their coding potential and differentially colored on the top. Number of insertions located inside coding exon is indicated. (**I**) Validation results for 51 insertions identified above and 19 deletions identified within long reads by PCR. Shown are the percentages of validated indels that are detected using three different sources of DNA samples as color coded. The numbers of the validated events are indicated above each bar. N2_1 and N2_2 are two N2 *C. elegans* strain that have been independently maintained over 20 years. CB4856 is the Hawaii isolate of *C. elegans*. (**J**) Validation results of three types of insertion as identified in (**A**, **B** and **C**) by NGS data (see Methods). The number of the validated events is indicated above each bar. NA, not applicable.

A particular strength of the long reads lies on its capability to identify indels that are relatively big in size, which would be impossible or difficult using the short reads generated with existing NGS platforms. To evaluate the potential of the long reads in recovery of big indels, we first focused on recovery of indels that are longer than 9 bp, (a default cutoff set by CLC Workstation algorithm for variation calling) within individual long reads. We were able to detect 2109 deletions and 274 insertions that were longer than 9 bp within the long reads relative to the reference genome (Fig. 5A; Supplementary Table S4, S5). Because most of the identified indels were supported by multiple independent reads, we decided to examine whether these indels represented errors in *C. elegans* reference genome, or in the read or a product of spontaneous mutation uniquely accumulated in our N2 strain. To distinguish these possibilities, we performed PCR using the genomic DNAs from the following three sources as a template to validate a subset of the identified indels (see Methods). The genomic DNAs were extracted from our N2 (N2_1), the second N2 that has been separately cultivated from ours for at least 20 years (N2_2) and the Hawaii isolate of *C. elegans s*train CB4856. To our surprise, 42 out of the 47 tested insertions were confirmed to be present in all three sources whereas only 3, 1 and 1 out of the 17 tested deletions were confirmed to be present in N2_1, N2_2 and CB4856 respectively (Fig. 5E; Supplementary Table S6 ). Presence of insertions in the reads that are confirmed by the PCR product derived from all three sources of *C. elegans* isolates indicates that the relevant sequences are missing in the current *C. elegans* genome.

In contrast, most of the deletions in the reads were not supported by the PCR results. The high false-positive rate of the deletions in reads suggests an intrinsic issue associated with the pre-assembling of the long reads from the raw shorts reads. Consistent with this, most of the deleted sequences within reads tend to contain repetitive sequences (Supplementary Table S3). The elevated false-positive rate of deletion in reads also explains the five-time higher frequency of the observed deletion than that of the insertion and SNV (Supplementary Figure S4). Taken together, it is the presence of certain type of the repetitive sequences especially those positioned in tandem that not only prevents pre-assembly of a long read with sufficient length, but also contributes to the observed higher error rate of deletion in the long reads relative to the reference genome.

### Recovery of sequences missing in the current *C. elegans* genome using the reads with unaligned ends

Given the high degree of accuracy in detecting sequences missing in the reference genome with the long reads, we further explored the possibility of detecting such missing sequences within a single or between two adjacent long read(s) but with a more accommodating alignment method (Figs. 5B,D). Specifically, if a long read can be aligned against the reference genome at one arm but not the other, which is called an unaligned end, the alignment parameters were relaxed so that both arms of the read can be aligned locally against the reference genome except for the mid-part of the read, which is referred to as “Self-mapped” (see Methods). The alignment parameters were relaxed because the initial alignment was made with a high stringency, i.e., demanding 99% identity within a continuous one Kbp region between the query and the reference sequences. To enable the insertions to be recovered by the two adjacent reads that were able to be partially aligned against the reference genome at their one arm but not the other (unaligned end), the possible insertion can be recovered by their overlapping unaligned ends (“Cross-mapped”) (Fig. 5C) or non-overlapping unaligned ends (“Close breakpoint”) (Fig. 5D).

In addition to the 274 insertions (> = 9 bp in size) identified within individual reads using the stringent alignment parameters stated above, another 44, 8 and 75 insertions were recovered with “Self-mapped”, “Cross-mapped” and “Close breakpoint” respectively. A total of 28 identified missing sequences are present within an existing protein-coding exon whereas the remaining ones are located within the intronic or intergenic regions (Fig. 4, Supplementary Table S7).

**Figure 6:**
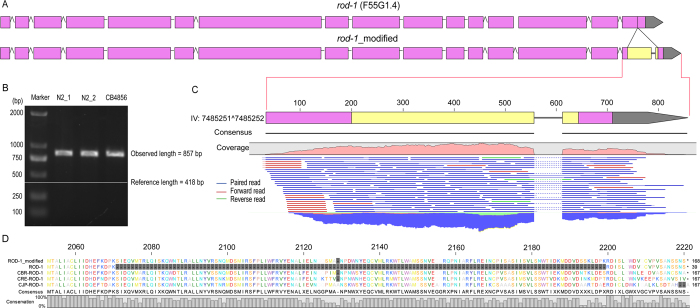
A revised gene model of ***rod-1*** and its validation based on an insertion located within its coding exon. (**A**) A revised gene model of *rod-1* with an insertion that alters its last exon. Pink, existing exon, yellow, newly added exon. (**B**) Validation of the insertion by PCR with three sources of DNAs as indicated in 5(**I**). Shown also are the observed and expected sizes of the PCR products. (**C**) A magnified view of the affected exon that is confirmed by RNA-seq data whose mapping reads are shown below the corrected gene model. (**D**) Partial multiple alignment using the *C.elegans* ROD-1 protein sequence and its orthologs in *C. briggsae* (CBR), *C. remanei* (CRE) and *C. japonica* (CJP). Discrepancies in alignment are shaded in grey.

To validate the identified insertions using independent method, the existing genomic or RNA-seq NGS data were mapped against the identified insertions along with its 2.5-Kbp flanking sequences within the reads (see Methods). Over 85% of the insertions can be confirmed by the genomic sequencing data (Fig. 5F). 14 out of the 21 insertions located within an existing protein-coding exon can be fully or partially validated with the RNA-seq data (Fig. 5; Supplementary Table S7). To illustrate the impact of the insertion on the existing gene model, an insertion located within a protein-coding exon and intron were chosen for gene model repairing respectively (Fig. 6; Supplementary Figure S6). Inclusion of an insertion of 439 bp between position 7485251 and 7485252 on chromosome IV led to a change in the existing gene model of *rod-1*, i.e., expanding its last exon and splitting it into two separate ones (Fig. 6). The missing fragment was not only confirmed by PCR (Fig. 6B), RNA-seq (Fig. 6C), but also by the multiple alignment of protein sequences among different nematode species (Fig. 6D). Consequently, a total of 129 missing amino acids were recovered relative to the existing ROD-1 protein sequence. Inclusion of another insertion in the first intron of *sul-2* between positions of 8158794 and 8158795 on chromosome V produced a new exon, which was confirmed by RNA-seq and multiple alignment of protein sequences from different nematode species (Supplementary Figure S5). As a result, a total of 106 missed amino acids in the current SUL-2 protein sequence can be recovered. At least 40 Kbp missing fragments can be recovered by the long reads and independently confirmed PCR and/or existing NGS data (Supplementary Table S8).

### Usefulness of the long reads in *de novo* genome assembly

**Figure 7:**
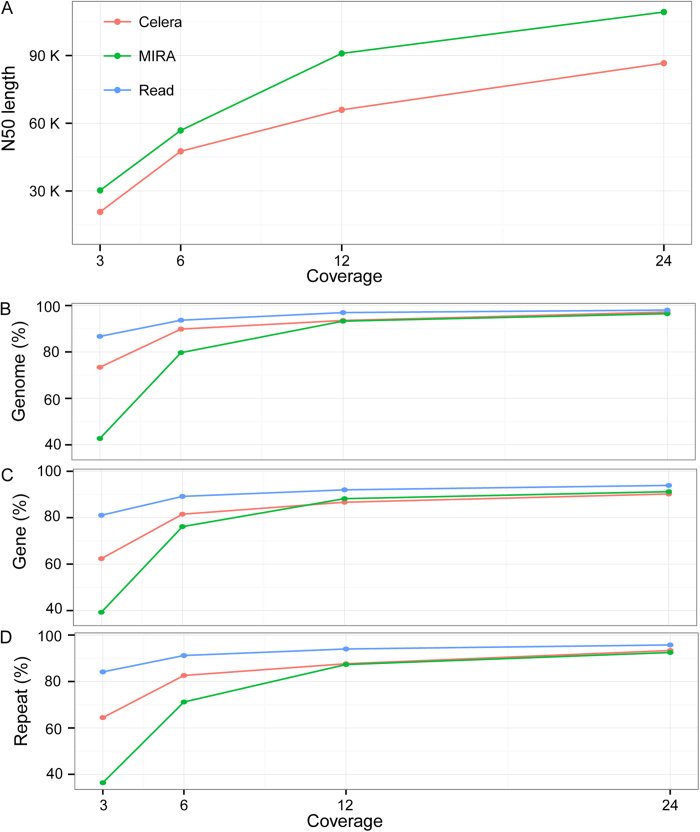
Effect of sequencing depth on ***de novo*** genome assembly using two genome assemblers. (**A**) Plotting of contig N50 length against the sequencing depth. (**B**, **C** and **D**) Plotting of read (blue) and contig (red and green) coverage of genome, protein-coding genes and repetitive sequences against the sequencing depth respectively. Contigs were assembled using Celera (red) or MIRA (green) assembler with 3×, 6×, 12× and 24× reads.

To test the usefulness of the long reads in genome assembly, we performed *de novo* assembly of *C. elegans* genome with a 3×, 6×, 12×and 24×coverage of the long reads respectively using two different Overlap Layout Consensus (OLC) based assemblers, Celera and MIRA^18^. The contigs assembled with Celera appears to be more accurate than those assembled with MIRA in terms of misassembling errors, including relocation, translocation and inversion (Table 1). However, the contigs assembled with MIRA have a longer N50 length while the achieved genome coverage by the assembled contigs is comparable between the two assemblers (Fig. 7; Table 1). Assembly with both algorithms demonstrated that genome coverage by the contigs plateaued with approximately 12×coverage of the reads, which represents approximate 93% of the *C. elegans* genome (Fig. 7B). However, the N50 length can still benefit substantially from the increasing read coverage from 12×to 24×(Fig. 7A), indicating a higher read coverage is needed for *de novo* assembly of a genome with a high quality, especially for those genomes that are big in size or contain substantial heterozygosity. Notably, the contigs produced with both assemblers suffer from a relatively high misassembling rate (Table 1).

Given the bias of the read sequence and a relatively small N50 contig size as well as a high rate of misassembling errors associated with the long reads compared with that of other long reads such as PacBio^19^, complement of Illumina long read sequencing by independent sources of reads, for example from PacBio and/or mate-pair sequencing is necessary to generate a “finished” grade of genome.

## Discussion

### A finished isogenic genome with resolved repetitive sequences allows an unbiased assessment of read accuracy

The estimated error rate of the Illumina long-reads using *Drosophila melanogaster* genome^8^ differs substantially from ours. For example, their SNV rate is about four times higher than ours (0.051% vs 0.011%) while their deletion rates is about one quarter of ours (0.029 vs 0.101%) respectively, though the insertion rates are comparable (0.019% vs 0.017%). One plausible reason underlying the observed discrepancy is that a repeat-masked genome was used for the data analysis in the previous study while an unmasked complete genome containing all types of repetitive sequences was used in this study. For example, our deletion rate in reads is 0.0017% if the mapping was performed with a repeat-masked *C. elegans* genome (around 14 Mbp masked, see Methods), which is comparable to that reported with *D. melanogaster*. Inclusion of all types of repetitive sequences allows an unbiased and systematic assessment of the read accuracy and mappability. Another possible reason underlying the elevated SNV rate observed in *D. melanogaster* genome is likely due to the sequence uncertainties associated with heterozygosity that have been sequenced with a lower confidence^20^, whereas hermaphroditic mode of reproduction produces an isogenic genome in *C. elegans*. In addition, a likely higher error rate in *D. melanogaster* reference genome than that in *C. elegans* may also contribute to the elevated error rate in the former species.

### The long reads hold promise for repairing even the “finished” grade genome

The short reads generated by NGS platforms have been widely used in genomic variation calling between wild isolates of the same species including *C. elegans*^10^. They have also been used to detect possible errors in reference genomes. For example, sequencing of an N2 sister strain, LSJ1 with read length of 36 bp has identified an insertion of 34 bp in reads that is mapped to the genomic position of 3568818 and 3568819 of chromosome II^17^. Alignment of our long reads against the *C. elegans* genome extends the 34-bp insertion into its full size of 339 bp, demonstrating the potential of the long reads in recovering the genomic fragments that are relatively big in size but missing from the reference genome, which would be difficult with the short reads. We expect the long reads will be useful in refining the draft genomes of other *Caenorhabditis* species, especially those of *C. briggsae* and *C. nigoni*, that have recently been developed as a model for study of speciation genetics^21^,^22^.

### Novel assembling algorithm is needed to accommodate the long reads in *de novo* genome assembly

OLC based assemblers are expected to accommodate the long reads better in *de novo* genome assembly than the de Bruijn graph-based ones, which is likely due to the limitation on the K-mer size imposed by the latter^23^. For example, the N50 size for the contigs produced by MIRA and Celera using 24 × reads is 109.3 and 86.6 Kbp respectively without any scaffolding steps (Table 1) while the N50 size is only around 22 Kbp for the contigs produced using de Bruijn graph-based assembler in CLC Genomic WorkBench with the same 24 × reads in *C. elegans* (our unpublished data). However, the contigs produced by MIRA and Celera still suffer a relatively high mis-assembly rate. The most common errors include relocation and translocation. To explore the potential causes underlying the observed errors, we pulled out a typical translocation event, in which a fragment from chromosome V was incorrectly merged with another one from chromosome I (Supplementary Figure S6A). First, there is an apparent error in pre-assembly of the read that spans the two fragments, which is caused by two 1.2 Kb-repeats that are nearly identical in sequence and located at the opposite ends of each contig (Supplementary Figure S6A). Second, about half of the long reads can still be correctly preassembled so that they are able to span the repetitive regions correctly (Supplementary Figure S6B, S6C). However, Celera assembler tends to neglect these reads that correctly span the boundary between the two contigs but decides to use the single incorrectly assembled read that is substantially longer than the correctly assembled ones to extend the contig. This is because the assembler chooses the read with the longest overlapping region instead of the one with a higher coverage but a shorter overlapping size. This type of error is expected to be fixed by fine-tuning the weighting between read overlapping length and coverage in the assembling algorithm.

In summary, the Illumina synthetic long reads are highly accurate and able to recover most types of repetitive sequences although the reads of different lengths show certain coverage bias against target regions with different sequence contents. The reads would be invaluable in genome finishing, including recovery of missing sequences and/or detection of sequence rearrangement in a draft genome. Tandem arrangement of short repeat unit prevents preassembly of reads with sufficient length using HiSeq raw reads, especially when the region consisting of the tandem repeat is longer than 500 bp, which results in failed recovery of these genomic regions by the long reads. *De novo* assembly with the long reads demonstrates their use in genome assembly, but also highlights a need of novel genome assembling algorithm to accommodate the long reads.

## Methods

### *C. elegans* strains

N2 strain from our own lab (shipped from Seattle, WA, USA in 2010) was used for extraction of genomic DNA and sequencing library preparation. For PCR validation of the detected indels, genomic DNAs from three different lines of *C. elegans* were used as a template, including our own N2 strain (N2_1), another N2 strain that has been independently cultivated for at least 20 years from our N2 strain obtained from a local lab (N2_2) and a Hawaii isolate of CB4856.

### Library preparation, sequencing and read pre-assembly

The libraries were prepared according to Illumina’s protocol. Briefly, 500 ng high quality gDNA was sheared by g-Tube (Covaris). The sheared gDNA went through end repair, dA-tailing and adaptor ligation. The ligated products were size-selected for 8–10 Kbp range by agarose gel and eluted followed by quantification with qPCR. Based on qPCR quantification, the long insert library was diluted and distributed into a 384-well plate, with each well containing about 3 fg of the library. After long range PCR, the PCR product went through Nextera tagmentation and barcoding with 384 different barcoding PCR primers. The final products were pooled together, cleaned, concentrated and size-selected. A total of two final libraries were made using the same long insert library mentioned above. Each library was sequenced by a HiSeq 2500 Rapid Run Flowcell at 2 × 101 bp with 8-bp barcode. The primary sequencing results were streamed to BaseSpace and pre-assembled using the TruSeq Long-Read Assembly App v1.0 (https://basespace.illumina.com/). The application first pre-processed the reads to correct the sequencing and PCR errors and normalized read depths across fragments. In the second stage, a string graph was constructed using the String Graph Assembler (SGA); the resulting graph was then cleaned using the paired-end information from the short reads to produce an initial set of contigs. In the third stage, the contigs were subjected to further scaffolding to resolve repeats and fill the gaps that were created due to low sequencing coverage. In the final stage, the scaffolds were examined for possible sequence errors and mis-assemblies, and corrected. The assembled long-reads were generated as FASTQ files that were used in the subsequent analysis.

### Read decontamination and mapping

To filter out the possible DNA contaminations from other organisms in the reads, BLASTN was run locally against NR database with default parameters using all reads as an input to identify the potential contaminations. The hits with best match (smallest e-value) to a target that does not belong to *C. elegans* genome were treated as contaminated reads and thus removed from the downstream analysis (Supplementary Figure S1).

To map the reads against reference genome, *C.elegans* genome was downloaded from WormBase (WS242)^16^. All the reads bigger than 1.5 Kbp in size were mapped against the reference genome using BWA-MEM using the following parameters, i.e., single-ended, minimum seed length of 1000 bp and band width of 23 Kbp (the longest size of the reads)^24^. Approximately 0.07% (around three thousands) of the reads cannot align with the reference by BWA-MEM using the above parameters. To accommodate these reads, they were mapped again to the genome as stated above except with a minimum seed length as default. The resulting BAM files from two rounds of mapping were merged, sorted and indexed with SAMtools^25^, and the read coverage among chromosomes were calculated using BEDTools (v2.20.1)^26^. Given the significance of mapping method in variation finding, for example, mapping of the long reads using small seed length will generate small mapping segments and make mapping ambiguous, we employed 2 rounds of BWA-MEM mapping with different band size respectively to minimize the ambiguous mapping segments.

The *C. elegans* repetitive sequences were identified using the existing ones detected by RepeatMasker (http://www.repeatmasker.org), which defined 10.31% and 2.28% of the *C. elegans* genome as interspersed and locally repetitive sequences. The former can be further divided into seven main classes, including DNA repeat, LINE, SINE, LTR, RC/Helitron, DNA like repeat and some unknown repeats. The latter can be further divided into simple repeat, satellite repeat and low complexity region. The gene and tandem repeat annotations were retrieved from WormBase^16^. Only annotations for 20487 protein-coding genes were used for analyzing the gene coverage. A repeat or a gene was defined as “recovered” by the long reads when the full length of its sequence is covered at least once by a single or overlapping read(s).

### Variation calling

The positions of SNVs and indels identified within reads relative to the reference genome were output by parsing the CIGAR string and MD tag from the mapping BAM files. To characterize the genomic distribution of the variations between the long-read sequences and the reference genome, the mapping BAM files were parsed using “probabilistic variation detection” tool in CLC Genomic Workbench 7.0.3, with the following parameters: the minimum coverage of 1, minimal variation count of 1, probability larger than 0.5 and ploidy of 2 (http://www.clcbio.com/support/white-papers/). The resulting variations in the long reads relative to the reference genome were classified into four categories, namely SNV, insertion, deletion, and complex variation. SNV was defined as a single nucleotide variation, meaning one base was substituted by another. “Insertion” was defined as an inserted sequence in reads relative to the reference genome, while the “deletion” as a deleted sequence in the long read relative to the reference genome.

The “probabilistic variation detection” tool of CLC Genomic Workbench can only recover insertion sequence within a single read. To recover the possible insertions that may be resolved by two long reads located adjacent to each other in the reference genome, the unaligned ends generated by the read mapping were parsed using the “indel and structure variation finding” tool in CLC Genomic Workbench. The “indel and structure variation finding” tool was used to locate the breakpoint of the alignment, and the identified unaligned end was mapped back to the reference genome locally. Alternatively, the local unaligned ends generated by multiple independent reads that were closest between each other in distance were mutually aligned. Using the two tools, four types of insertions can be recovered, i.e., the “Within read”, “Self-mapped”, “Cross-mapped” and “Close breakpoint”. Identical insertions recovered with two independent methods were merged to a single one.

### PCR validation of the identified indel that is longer than 40 bp in size

To validate the identified long insertions and deletions within or between long reads, a subset of 48 insertions and 17 deletions that are longer than 40 bp were validated using PCR (Supplementary Table S6). The presence or absence of an indel can be distinguished by examining the product size.

### Validation of the identified insertions using existing NGS data

Two datasets, DRR008443 and SRR1050780 were downloaded from Sequence Read Archive (SRA)^27^ to validate the insertions that are longer than nine bp in size. The former is the 70 × N2 genomic DNA sequencing data generated with HiSeq (paired-end, 2 × 100 bp, library size of 500 bp). The latter is the RNA-seq data of mix-staged N2 mRNA (paired-end, 2 × 75 bp, library size of 300 bp). All insertions along with its two flanking 2500 bp sequences were pooled into a single file to be used as a reference. The genomic DNA sequencing data were mapped against the reference with “mapping reads to reference” tool of CLC Genomic Workbench using default settings. If an insertion was fully covered at least 10×across both boundaries by the paired end reads, the insertion was marked as “confirmed”. RNA-seq data were mapped against the reference using “large gap read mapping” tool of CLC Genomic Workbench to validate the insertion located within an existing protein-coding gene model. The region with at least 2×coverage was treated as an exonic region. The gene model was re-annotated/repaired using the “gene prediction tool”. The protein coding sequences of *sul-2* and *rod-1* and their homologous proteins were downloaded from Wormbase^16^. Multiple sequence alignment was carried out with the CLC genomic workbench using the default settings.

### *De novo* genome assembly and contig assessment

The subset of reads were generated randomly from the combined FASTQ file of the two libraries containing 24×reads (length > 1.5 Kb) to give rise to 3×, 6×and 12×of long reads. Two overlap-layout-consensus (OLC) assemblers were used to assemble the genome region respectively. The major parameters for Celera (8.1)^28^ were ovlMinLen = 500, unitigger = bogart, ovlMerSize = 31, utgGraphErrorRate = 0.0045, utgMergeErrorRate = 0.003, merThreshold = auto*2. And the major parameter for MIRA (4.0.1)^18^ were -AL:mo = 500, -SK:bph = 31, -SK:pr = 99, -AL:mrs = 99, -CO:fnicpst = yes. To evaluate the characteristics of the contigs assembled with Celera (8.1) or MIRA (4.0.1), the prevalence of SNVs, indels and other mis-assembly events were calculated using the QUAST (2.3)^29^.

### Data availability

Sequence data can be found under the NCBI BioProject: SRP047457, BioSample: SRS708640 and Experiment: SRX709602. (SRA RUN No pending).

## Additional Information

**How to cite this article**: Li, R. *et al*. Illumina Synthetic Long Read Sequencing Allows Recovery of Missing Sequences even in the “Finished” *C. elegans* Genome. *Sci. Rep*. **5**, 10814; doi: 10.1038/srep10814 (2015).

## Supplementary Material

Supplementary Information

Supplementary Tables

## Figures and Tables

**Table 1:** Evaluation of the performance of *de novo* genome assembly using MIRA and Celera.

Assembly	MIRA	Celera
***Statistics of contig***		
Number of contigs	1,972	3,094
Total size of contigs (bp)	109,184,716	107,097,920
Largest contig (bp)	717,688	650,163
N50 contig length (bp)	109,277	86,600
L50 count	274	316
Contig GC content (%)	35.51	35.48
***Statistics of contig mapping***		
Genome coverage (%)	96.48	97.18
Duplication ratio	1.134	1.103
NA50 contig length (bp)	82,984	78,179
LA50 count	349	372
Relocations	443	225
Translocations	245	131
Inversions	40	28
SNVs per 100 Kb	24.6	19.52
Short indels (<9 bp)	0.01195%	0.00647%
Long indels (>=9 bp)	0.000143%	0.000049%
Fully unaligned contigs	0	8
Partially unaligned contigs	6	29

The N50 length measures the length of the contig for which 50% of the total assembly length is contained in contigs of that size or larger, while the L50 metric is the ranking order of the contig if all contigs are ordered from longest to shortest. NA50 and LA50 are similar to N50 and L50 respectively except they are based on the alignment of the contigs against the genome. The relocation is a mis-assembly event that a single contig is “broken” with a minimum interval size of one Kbp and can be mapped to different regions of the same chromosome, while the translocation is the mis-assembly event that a single contig can be mapped to different chromosomes. The inversion is a mis-assembly event that a contig can be aligned to the opposite strands of the same chromosome. Duplication ratio is defined as the ratio of contig length and reference length.
