# Supplementary material for: Illumina Synthetic Long Read Sequencing Allows Recovery of Missing Sequences even in the “Finished” C. elegans Genome
Source: Sci Rep. 2015 Jun 3;5:10814. doi: 10.1038/srep10814 (PMC4650653; doi:10.1038/srep10814)
Supplement: Supplementary Information [file srep10814-s1.pdf]

Supplementary Information

Illumina Synthetic Long Read Sequencing Allows Recovery of Missing Sequences even in the “Finished” *C. elegans* Genome

Runsheng Li[1], Chia-Ling Hsieh[2], Amanda Young[2], Zhihong Zhang[2], Xiaoliang Ren[1], Zhongying Zhao[1, 3, *]

[1]Department of Biology, Hong Kong Baptist University, 224 Waterloo Road, Kowloon Tong, Hong Kong, China, [2]Illumina Inc., 5200 Illumina Way, San Diego, 92122, USA, [3]State Key Laboratory of Environmental and Biological Analysis, Hong Kong Baptist University, Hong Kong, China

[*] To whom correspondence should be addressed: Zhongying Zhao

Email: zyzhao@hkbu.edu.hk

Phone: 852-3411-7058

This file includes:

Supplementary Figures S1 to S6 and the Figure legend

Supplementary Figure Legends

Supplementary Figure S1. Flowchart of data analysis.

Supplementary Figure S2. Differential coverage of genome or repetitive sequences by read of various lengths. (A) Histogram of read coverage on *C. elegans* chromosome III. Read length is scaled in grey as indicated. SNP density is shown on the top. Track of mapping gap is shown in black while those of repetitive sequences are differentially color coded and shown at the bottom. (B) A magnified view of the biggest gap in panel A which occupies a 47 Kb region consisting exclusively of satellite repeat as highlighted at the bottom. The aligned reads of both orientations are shown on each side of the gap. (C) Output of "dotplot" using the gap sequence in panel (B) as an input. Scale of sequence similarity is indicated on the top.

Supplementary Figure S3. Read coverage of ribosomal genes. (A) Read coverage of the ribosomal gene cluster consisting of 18S/5.8/28S genes currently placed at the end of chromosome I. Annotation of the gene cluster is shown at the bottom. Note, only a single copy of the ribosomal gene cluster was placed in the *C. elegans* reference genome. (B) Read coverage of the ribosomal gene 5S genomic region located on chromosome V. The red and blue bars indicate the unique mapping of forward and reserve reads, while the yellow bar indicates ambiguous mapping, meaning that a read was mapped to different genomic regions with equal efficiency.

Supplementary Figure S4. Density of deletion (red), insertion (green) and SNV (blue) events identified using the long reads over *C. elegans* chromosomes.

Supplementary Figure S5. Validation of a revised gene model based on an insertion that is located within an intron. (A) A revised gene model of *sul-2* with an insertion that adds a new exon. Pink, existing exon, yellow, newly added exon. (B) A magnified view of the affected exon that is confirmed by RNA-seq data with its mapping reads shown at the bottom. (C) Partial multiple alignment using the *C.elegans* SUL-2 protein sequence and that of its orthologs in *C.briggsae* (CBR), *C. remanei* (CRE) and *C. japonica* (CJP). Discrepancies in alignment are highlighted in grey.

Supplementary Figure S6. An example of translocation event found in the contig assembled by Celera. (A) Shown is the entire region of the contig assembled with two smaller contigs that are nevertheless derived from chromosome V (red thick bar) and I (blue thick bar) respectively. Chromosomal coordinates for the two fragments are indicated on the top. Note, the two are merged together by the Celera due to the single read which was preassembled incorrectly likely because of the overlapping sequences of around 1.2 Kb in size that contain nearly identical DNA repeats (thick yellow bar) between the two fragments. Reads used to assemble the “wrong” contig shown in panel A are drawn in thin bars. (B and C) Mapping of the incorrectly preassembled read (indicated by arrow) and its cognate genomic regions on chromosome V and I respectively with all other reads except for the wrong one. Note that the left and right arm of the read can align properly with its cognate genomic sequences respectively while the other arms remain unaligned (shown as wavy lines).

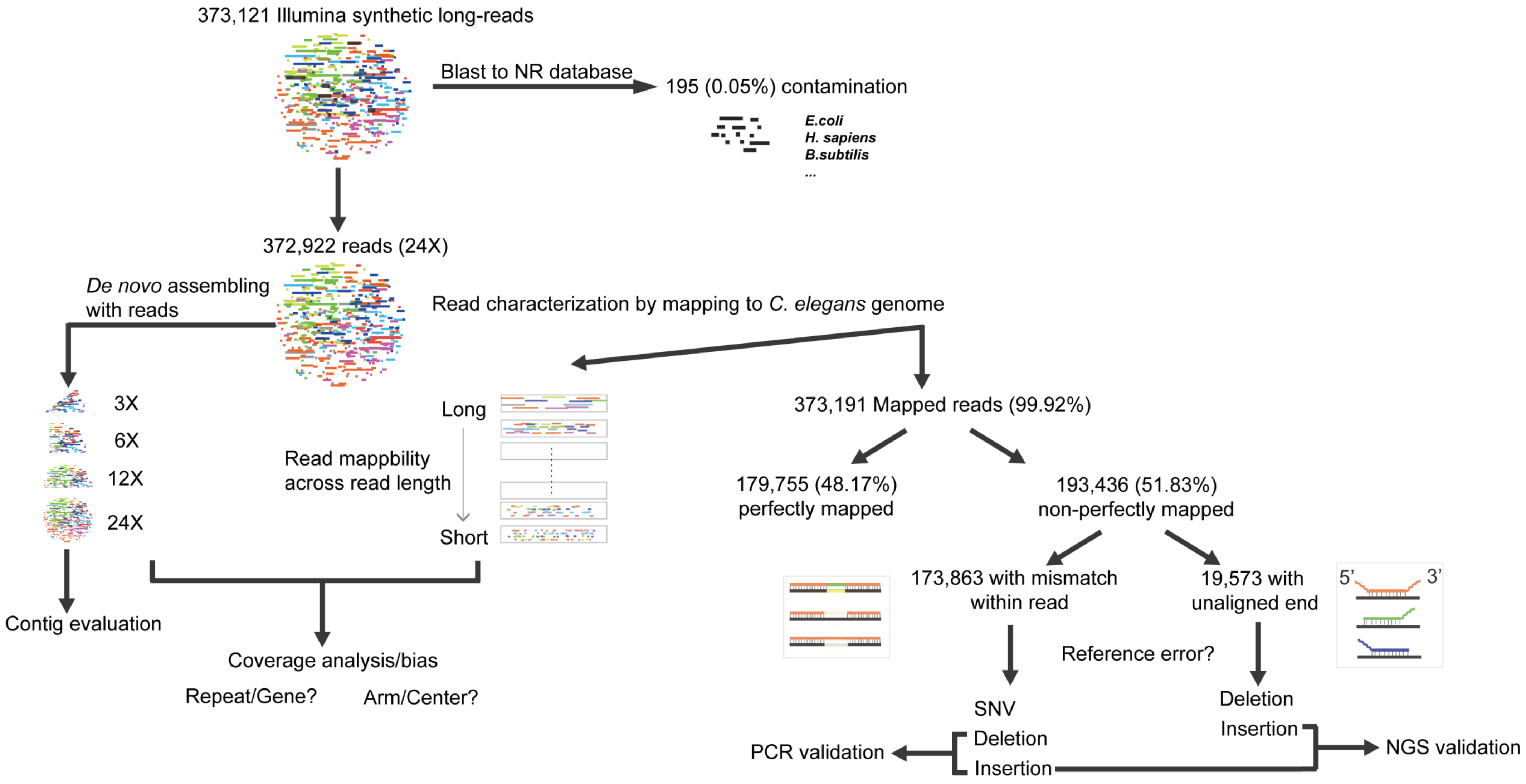
373,121 Illumina synthetic long-reads
Blast to NR database
195 (0.05%) contamination
E.coli
H. sapiens
B.subtilis
...
372,922 reads (24X)
De novo assembling with reads
Read characterization by mapping to C. elegans genome
3X
6X
12X
24X
Long
Read mappbility across read length
Short
373,191 Mapped reads (99.92%)
179,755 (48.17%) perfectly mapped
193,436 (51.83%) non-perfectly mapped
173,863 with mismatch within read
19,573 with unaligned end
5'
3'
Reference error?
Contig evaluation
Coverage analysis/bias
Repeat/Gene?
Arm/Center?
SNV
Deletion
Insertion
Deletion
Insertion
PCR validation
NGS validation

Figure S1

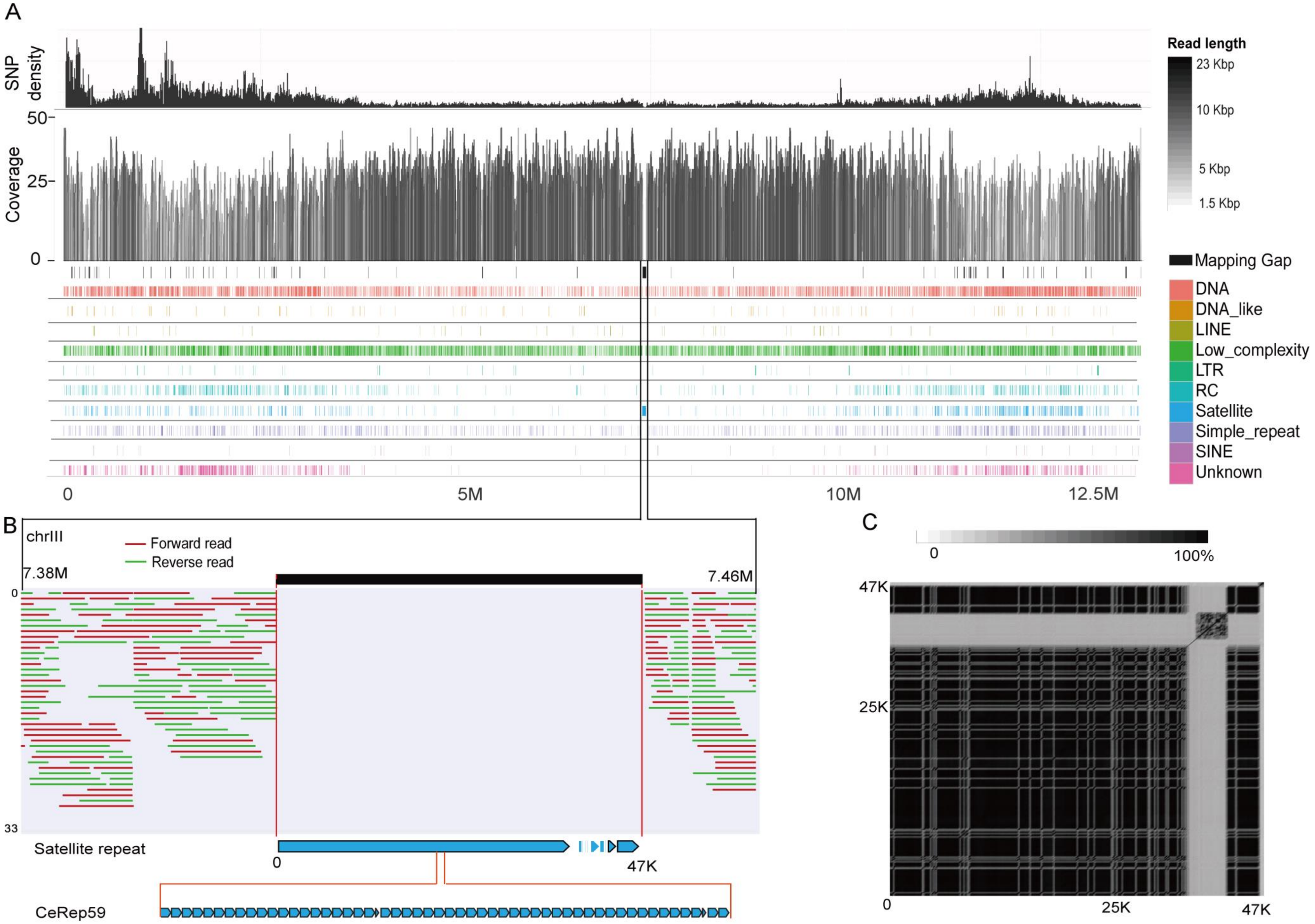
A
SNP density
Coverage
50
25
0
Read length
23 Kbp
10 Kbp
5 Kbp
1.5 Kbp
Mapping Gap
DNA
DNA_like
LINE
Low_complexity
LTR
RC
Satellite
Simple_repeat
SINE
Unknown
0
5M
10M
12.5M
B
chrIII
Forward read
Reverse read
7.38M
7.46M
0
33
Satellite repeat
0
47K
CeRep59
C
0
100%
47K
25K
0
25K
47K

Figure S2

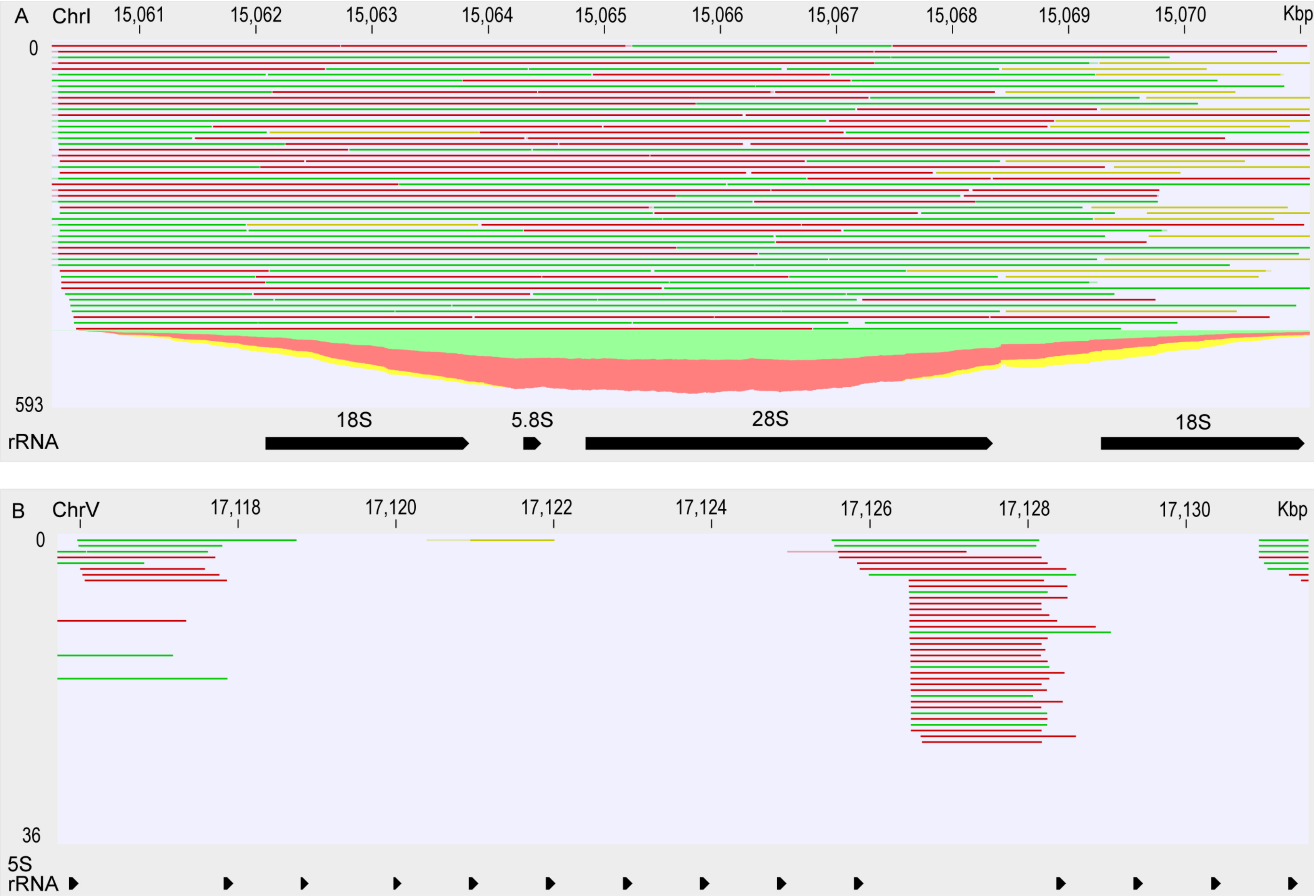

A
ChrI
15,061
15,062
15,063
15,064
15,065
15,066
15,067
15,068
15,069
15,070
Kbp
0
593
18S
5.8S
28S
18S
rRNA
B
ChrV
17,118
17,120
17,122
17,124
17,126
17,128
17,130
Kbp
0
36
5S
rRNA

Figure S3

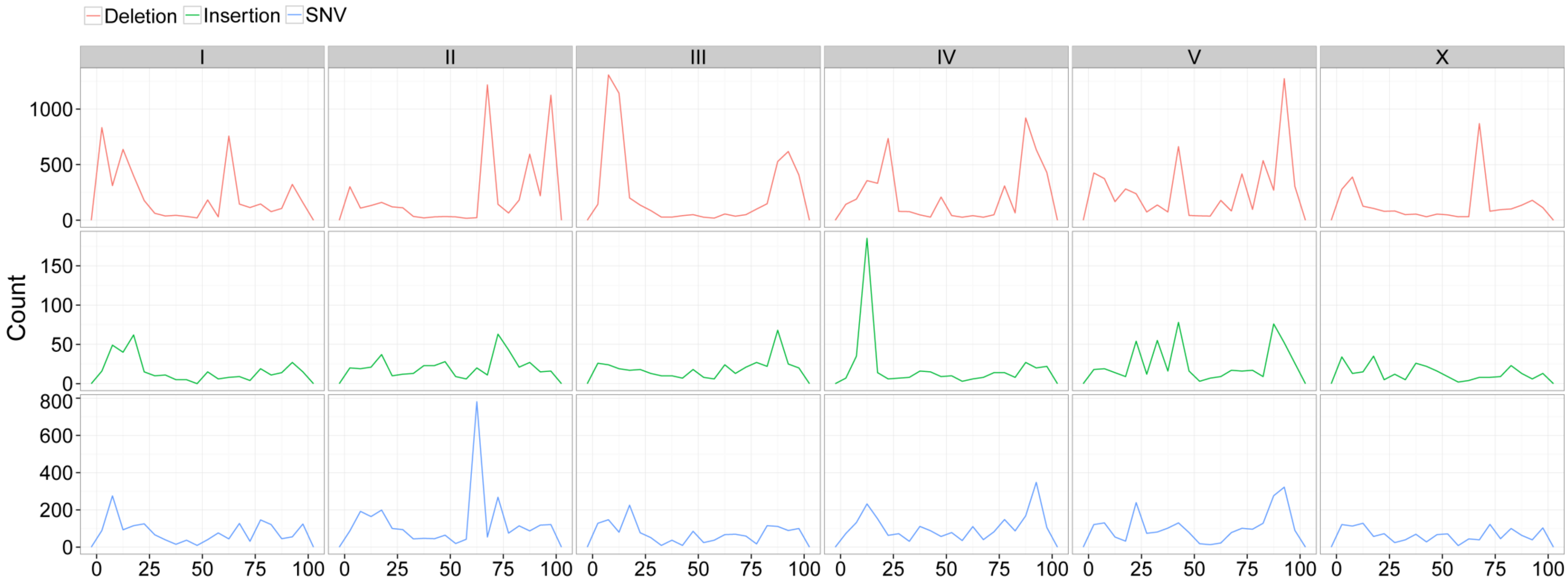
Deletion
Insertion
SNV
I
II
III
IV
V
X
Count
1000
500
0
150
100
50
0
800
600
400
200
0
0
25
50
75
100

Figure S4

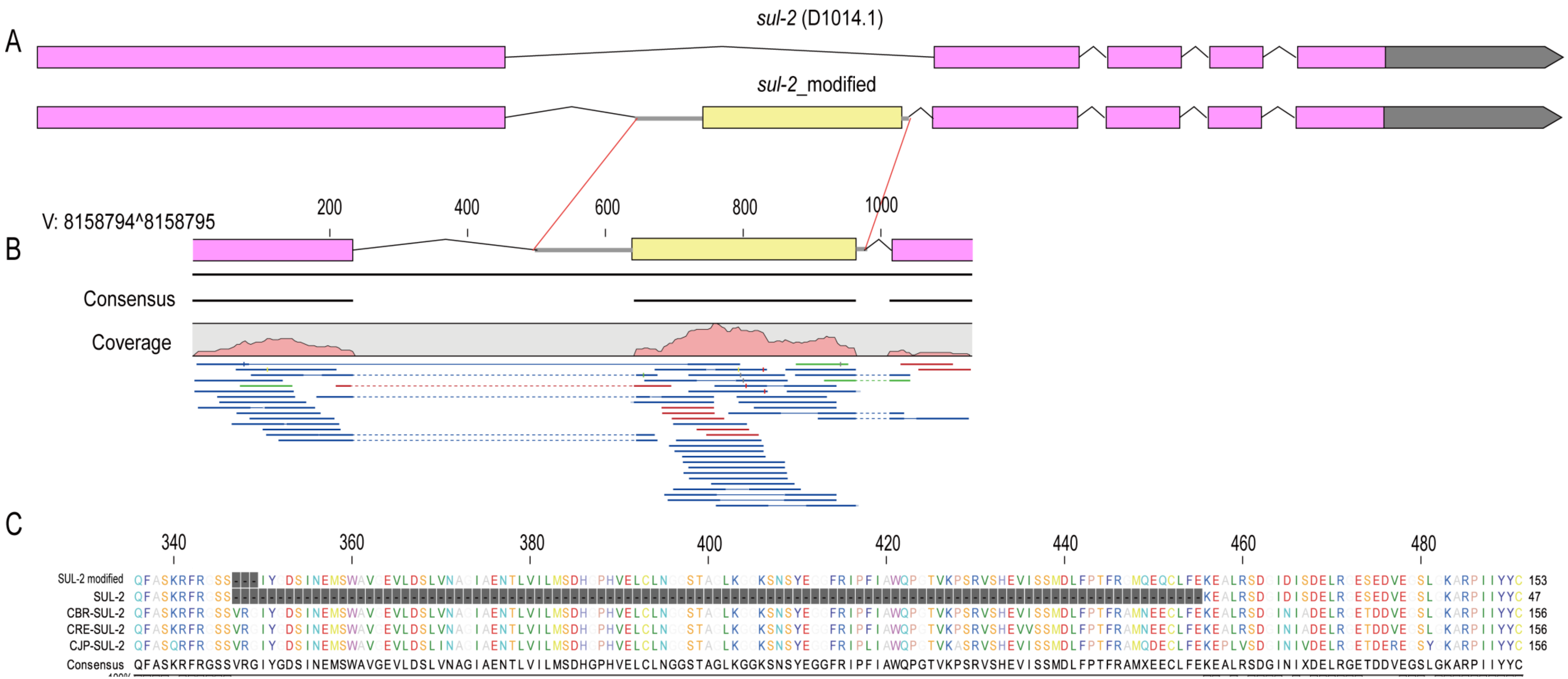
A
sul-2 (D1014.1)
sul-2_modified
B
V: 8158794^8158795
200
400
600
800
1000
Consensus
Coverage
C
340
360
380
400
420
440
460
480
SUL-2 modified
SUL-2
CBR-SUL-2
CRE-SUL-2
CJP-SUL-2
Consensus
153
47
156
156
156

Figure S5

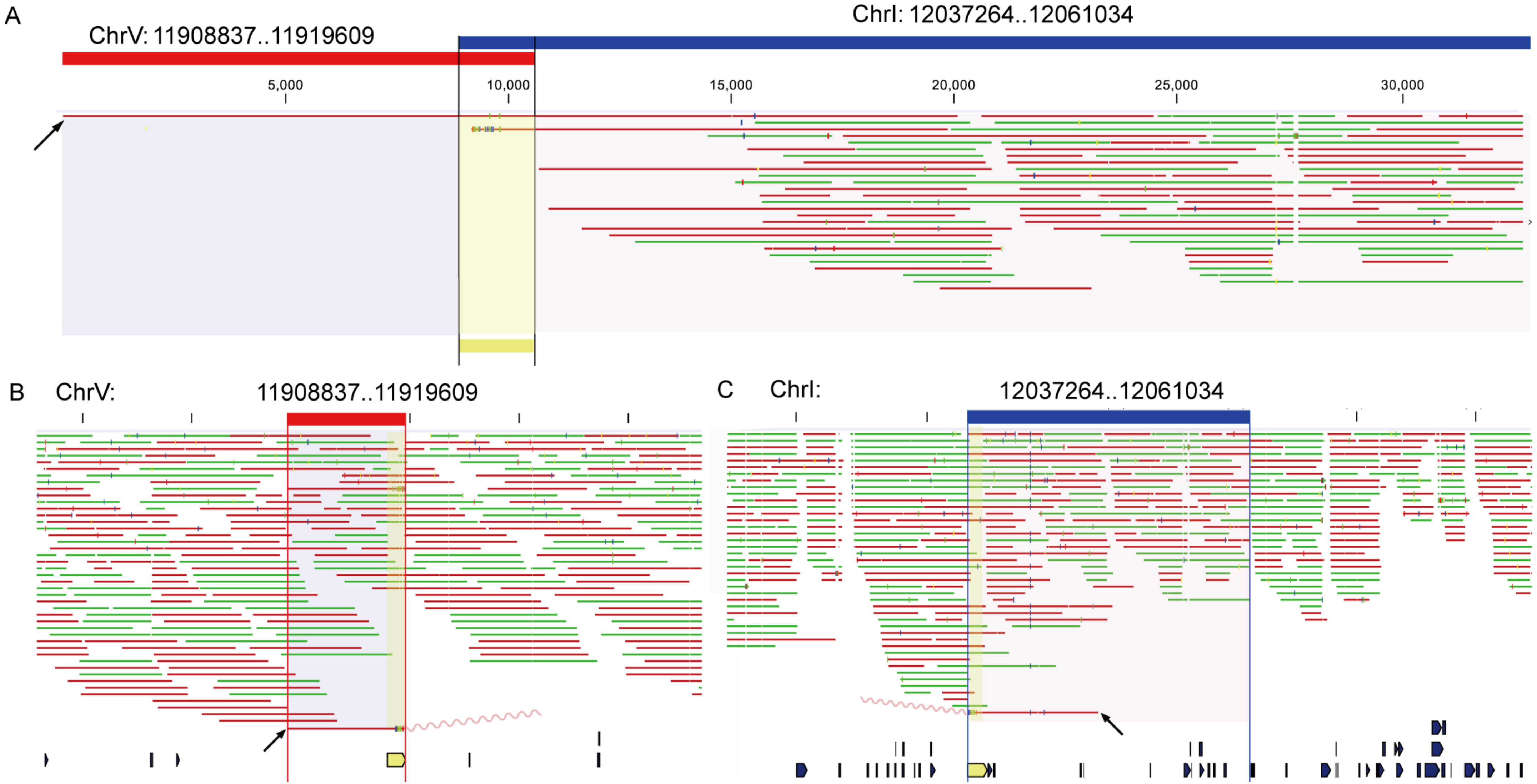
A
ChrV: 11908837..11919609
ChrI: 12037264..12061034
5,000
10,000
15,000
20,000
25,000
30,000
B
ChrV:
11908837..11919609
C
ChrI:
12037264..12061034

Figure S6
